# Case Report: Early documented infected pancreatic necrosis due to carbapenem-resistant *Acinetobacter baumannii* and concurrent *Enterococcus faecium* bacteremia: a salvage regimen with sulbactam-durlobactam and tigecycline

**DOI:** 10.3389/fmed.2026.1874666

**Published:** 2026-06-22

**Authors:** Man Li, Ying Fu, Ying Chen, Yunsong Yu

**Affiliations:** 1 Department of Critical Care Medicine, Sir Run Run Shaw Hospital, Zhejiang University School of Medicine, Hangzhou, China; 2 Zhejiang Key Laboratory of Precise Diagnosis and Treatment of Abdominal Infection, Sir Run Run Shaw Hospital, School of Medicine, Zhejiang University, Hangzhou, China; 3 Department of Clinical Laboratory, Sir Run Run Shaw Hospital, School of Medicine, Zhejiang University, Hangzhou, China; 4 Zhejiang Provincial Engineering Research Center of Innovative Instruments for Precise Pathogen Detection, Hangzhou, China; 5 Department of Infectious Diseases, Sir Run Run Shaw Hospital, Zhejiang University School of Medicine, Hangzhou, China

**Keywords:** abdominal compartment syndrome, carbapenem-resistant *Acinetobacter baumannii*, *Enterococcus faecium*, infected pancreatic necrosis, severe acute pancreatitis, sulbactam-durlobactam

## Abstract

**Objective:**

To report an early documented case of culture-proven infected pancreatic necrosis (IPN) caused by carbapenem-resistant *Acinetobacter baumannii* (CRAB) with concurrent *Enterococcus faecium* bacteremia, and to describe a salvage regimen combining sulbactam-durlobactam (SUL-DUR) with tigecycline.

**Methods:**

The clinical data, laboratory findings, and treatment course of the patient were retrospectively described, with a review of the relevant literature.

**Results:**

A 51-year-old male with severe acute pancreatitis (SAP) developed persistent organ failure, abdominal compartment syndrome, and acute kidney injury requiring continuous renal replacement therapy (CRRT). On day 5, blood cultures grew enterococci (both *E. faecalis* and *E. faecium* from a single set), and the patient’s refractory clinical status prompted anti-enterococcal therapy. On day 7, percutaneous drainage first documented IPN caused by CRAB, followed by CRAB bloodstream infection. Because *E. faecium* is intrinsically resistant to all carbapenems, a carbapenem could not be added as the partner drug for SUL-DUR. Instead, we used SUL-DUR (1 g/1 g q8h, adjusted for CRRT) together with tigecycline. On day 13, after 4 days of SUL-DUR treatment (initiated on day 9), the patient showed clinical improvement and microbiological clearance. On day 19, after completing a 10-day course of SUL-DUR with persistent clinical improvement and two consecutive negative blood cultures, the agent was discontinued. The patient also required neuromuscular blockade for refractory abdominal compartment syndrome and delayed laparoscopic necrosectomy. He was discharged on day 75 in stable condition.

**Conclusion:**

IPN can be confirmed within the first week of SAP in patients with persistent organ failure and sustained intra-abdominal hypertension, especially in the presence of multidrug-resistant organism colonization. For polymicrobial infections with CRAB and intrinsically carbapenem-resistant *E. faecium*, the combination of SUL-DUR and tigecycline is a rational alternative when a carbapenem cannot be used.

## Background

Severe acute pancreatitis (SAP), characterized by persistent organ failure (OF), is associated with significant morbidity and mortality (1). The development of infected pancreatic necrosis (IPN) is a pivotal event that drastically increases mortality risk, typically occurring after the second week of illness (1–3). The co-occurrence of IPN with abdominal compartment syndrome (ACS) creates a vicious cycle of worsening splanchnic hypoperfusion, bacterial translocation, and organ dysfunction (4–6).

This clinical challenge is further exacerbated by the global dissemination of multidrug-resistant organisms (MDROs), particularly carbapenem-resistant *Acinetobacter baumannii* (CRAB), a WHO Priority 1 critical pathogen (7, 8). Polymicrobial infections involving both CRAB and *Enterococcus faecium* present a specific therapeutic dilemma. The 2024 Infectious Diseases Society of America (IDSA) guidance recommends Sulbactam-Durlobactam (SUL-DUR) combined with a carbapenem as the preferred regimen for CRAB infections (7). However, *E. faecium* exhibits high-level intrinsic resistance to all carbapenems mediated by low-affinity penicillin-binding protein 5 (PBP5) (9), rendering that combination ineffective against an established enterococcal infection.

We present a complex case of SAP, reported in accordance with CARE guidelines (10), that successfully navigated three converging crises: (1) early documented IPN confirmed on Day 7; (2) polymicrobial infection with CRAB and *E. faecium*; and (3) refractory ACS managed with neuromuscular blockade. This case highlights a critical gap in current antimicrobial guidance and provides a pragmatic, mechanism-based approach to regimen selection in polymicrobial infections.

## Case presentation

A 51-year-old male (height 168 cm, weight 70 kg, BMI 24.8 kg/m^2^) with a history of chronic alcohol abuse (approximately 50 g spirits daily for over 10 years), recurrent pancreatitis, and prior cholecystectomy was transferred to our intensive care unit (ICU) on day 2 of illness. No relevant family history was reported. The patient presented with acute abdominal pain, high-grade fever (39.8 °C), and signs of systemic toxicity. On admission, he was critically ill with a heart rate of 150 beats/min and required norepinephrine support (0.15 μg/kg/min) for shock. He was intubated and mechanically ventilated (FiO₂ 0.75, PEEP 12 cm H_2_O) for respiratory failure. Physical examination revealed a markedly distended and tense abdomen. Intra-abdominal pressure (IAP), measured via an intravesical Foley manometer in accordance with World Society of the abdominal compartment syndrome (WSACS) guidelines, was 22.1 mmHg, consistent with ACS (4).

Admission laboratory tests revealed lipase >2000 U/L, procalcitonin 27.26 ng/mL, interleukin-6 853 pg/mL, and creatinine 245 μmol/L (eGFR <30 mL/min/1.73 m^2^, KDIGO Stage 3 AKI), indicating SAP, profound systemic inflammation, and acute kidney injury (AKI). The patient received comprehensive organ support, including lung-protective mechanical ventilation, continuous renal replacement therapy (CRRT; continuous veno-venous hemodiafiltration with an effluent rate of 30 mL/kg/h), empirical antibiotic therapy with piperacillin-tazobactam (4.5 g q8h), and vasopressor support with fluid resuscitation.

Blood cultures drawn on day 2 grew both *Enterococcus faecalis* and *E. faecium* from a single bottle on day 5. Identification and susceptibility testing were performed using VITEK MS and VITEK 2 Compact (Bio-Mérieux, France) per CLSI M07/M100 guidelines. Although single-set isolation raised possible contamination, the patient had deteriorated after 3 days of piperacillin-tazobactam: persistent high fever, markedly elevated procalcitonin, and ongoing ACS (IAP 19–22 mmHg) (Table 1). Unable to exclude an enterococcal source, we initiated empirical therapy targeting both species without waiting for repeat cultures. At that time, CRAB was isolated only from sputum without invasive infection. Tigecycline was chosen for its excellent activity against both enterococci (MIC <0.12 for each), high intra-abdominal penetration, and no CRRT adjustment. A loading dose of 100 mg IV was followed by 50 mg q12h, along with contact isolation and chlorhexidine bathing. A second blood culture set (drawn day 5, positive day 8) grew only *E. faecium*, confirming *E. faecium* bacteremia, while *E. faecalis* was isolated only once.

**Table 1:** Clinical course and laboratory findings.

Day	2	3	4	5	6	7	8	9	10	11	12	13	—	19	—	42	—	75
Clinical events	ICU Adm			Blood cx + *E. faecalis + E. faecium*		CT-PAD	Blood cx + *E. faecium*	Blood cx + CRAB		NMBA			—		—	Laparoscopic debridement	—	Discharge
IAP (mmHg)	22.1	20.2	22	19	23	22	24.3	26	29	33	21	18	—	16	—	12.5	—	—
24 h fluid I/O (mL)	4845/554	3833/3264	2690/3228	2743/2935	3015/3623	2285/5463	3414/4459	3187/3776	4205/2571	4783/7566	4339/7783	2680/4678	—	3008/6350	—	3286/3005	—	—
hsCRP (mg/L)	414.5	355.1	379.3	176.7	229.7	186.7	311.7	362.9	375.7	271.4	267.9	233.5	—	119.6	—	74.7	—	43.2
PCT (ng/mL)	27.26	47.08	46.38	47.65	20.03	8.25	11.06	10.62	7.12	6.3	4.7	2.36	—	1.68	—	0.36	—	0.15
IL-6 (pg/mL)	853	884	198.2	338.1	105.9	256.2	2,583	1,246	2,903	>5,000	4,063	2,984	—	249.2	—	141.7	—	—
WBC (×10^9^/L)	10.6	11.5	12	16.4	22.3	30.9	26.4	19.4	22.3	22.0	21.9	23.1	—	18.6	—	14.5	—	11.1
T (°C)	39.8	38.8	38.5	38.7	38.6	39.6	38.8	36.6	37.4	37.6	37.9	36.8	—	38.2	—	37.2	—	36.8
Piperacillin-tazobactam	+	+	+	—	—	—	—	—	—	—	—	—	—	—	—	—	—	—
Tigecycline	—	—	—	+	+	+	+	+	+	+	+	+	—	+	—	+	—	—
Cefoperazone-sulbactam	—	—	—	—	—	+	+	—	—	—	—	—	—	+	—	+	—	—
Sulbactam-durlobactam	—	—	—	—	—	—	—	+	+	+	+	+	—	—	—	—	—	—

IAP, intra-abdominal pressure; I/O, input/output; hsCRP, high-sensitivity C-reactive protein; CRAB, carbapenem-resistant *Acinetobacter baumannii*; PCT, procalcitonin; IL-6, interleukin-6; WBC, white blood cell; T, body temperature; ICU, intensive care unit; Adm, admission; cx, culture; CT-PAD, CT-guided percutaneous aspiration and drainage; NMBA, neuromuscular blocking agent; +, administered on that day; —, not applicable/not available.

Abdominal contrast-enhanced CT (CECT) on day 5 confirmed acute necrotizing pancreatitis with extensive peri-pancreatic and pelvic fluid collections, without perforation (Figure 1).

**Figure 1:**
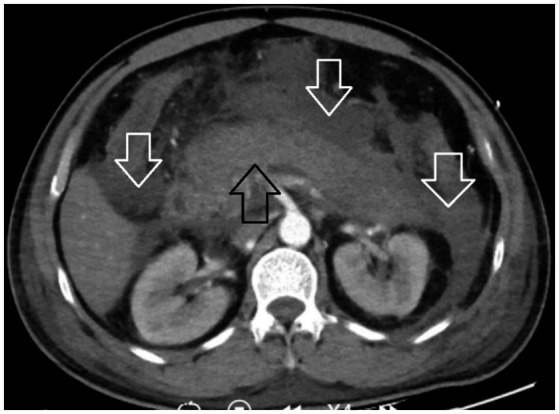
CECT day 5. Axial CECT image demonstrating acute necrotizing pancreatitis. Black arrow indicates enhancing pancreatic parenchyma, estimated necrosis at <30% of the glandular volume based on the absence of enhancement in the tail. White arrow indicates an acute peri-pancreatic necrotic fluid collection (ANC) extending into the greater omental sac and bilateral paracolic gutters.

Despite tigecycline therapy, the patient continued to experience recurrent fever and IAP > 20 mmHg. Suspecting intra-abdominal infection, on day 7, CT-guided percutaneous drainage was performed, placing four catheters. Gram stain of aspirated fluid from two separate sites revealed Gram-negative bacilli, later identified as CRAB. Since SUL-DUR was not immediately available, cefoperazone-sulbactam was initiated empirically to provide timely anti-CRAB coverage through its sulbactam component.

On Day 9, the patient developed CRAB bloodstream (blood drawn on day 8) infection with septic shock. The isolation of CRAB from two separate drainage sites and bloodstream necessitated the addition of SUL-DUR. The 2024 IDSA Guidance recommends combining SUL-DUR with a carbapenem to optimize anti-CRAB activity (7). However, that guidance was developed for the treatment of mono-microbial CRAB infections. In this patient, switching to a carbapenem would have withdrawn coverage for the pre-existing *E. faecium* bacteremia because of its intrinsic carbapenem resistance (meropenem MIC >8 mg/L). Therefore, we retained tigecycline as the partner drug for SUL-DUR, preserving activity against the full spectrum of pathogens. SUL-DUR (sulbactam 1 g plus durlobactam 1 g every 8 h, administered as a 3 h infusion) was added to tigecycline regimen (50 mg q12h). Dosing rationale during CRRT: Given the CRRT effluent rate of 30 mL/kg/h (estimated creatinine clearance approximately 42 mL/min), the q8h interval was selected based on *ex vivo* pharmacokinetic modeling data, which suggest that this regimen achieves a probability of target attainment (PTA) exceeding 90% for CRAB isolates with SUL-DUR MICs ≤4 mg/L (11). Therapeutic drug monitoring was not available.

IAP increased progressively, peaking at 33 mmHg on day 11. After failure of conservative measures (gastrointestinal decompression, pro-kinetics, enemas, traditional Chinese medicine, acupuncture, and repeated drainage on days 7 and 11), a continuous infusion of rocuronium was initiated as rescue therapy (bolus 40 mg, then 5 mg/h, approximately 1.4 μg/kg/min). IAP decreased from 33 mmHg to 21 mmHg within 24 h.

On day 15, with IAP consistently below 20 mmHg, post-pyloric enteral nutrition was initiated via a nasojejunal tube. On day 19, after completing a 10 day course of SUL-DUR with sustained clinical improvement and two negative blood cultures, the regimen was de-escalated. No adverse reactions related to antibiotic treatment were found. Given the high cost and limited availability of SUL-DUR, tigecycline plus cefoperazone-sulbactam was used to maintain sulbactam pressure against the CRAB. The same day, renal function had recovered sufficiently to permit discontinuation of CRRT. Extubation was successful on day 26. Oral intake was gradually reinstated, and the patient resumed a full oral diet by day 35.

A subsequent infection flare occurred in association with organized peripancreatic necrosis (Figure 2). Laparoscopic debridement of peripancreatic necrotic tissue was performed on day 42, with placement of six double-lumen drains for continuous postoperative lavage. Peripancreatic necrotic tissue culture remained positive for CRAB.

**Figure 2:**
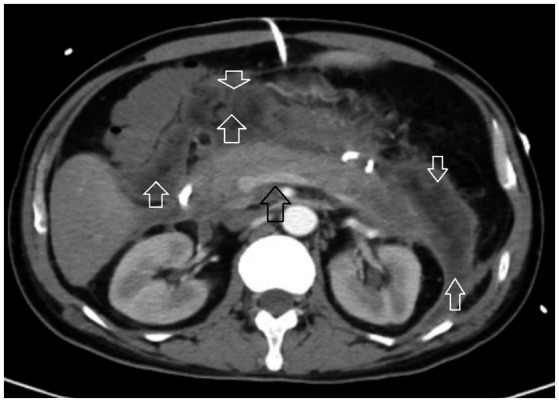
CECT day 36. Axial CECT image demonstrating evolution to walled-off necrosis (WON). Black arrow indicates residual pancreatic necrosis. White arrow indicates a well-defined peri-pancreatic encapsulated fluid collection with enhancing rim, consistent with walled-off necrosis.

### Outcome and follow-up

Following necrosectomy, inflammatory markers normalized, and follow-up imaging demonstrated a significant reduction in the peripancreatic collection (Figure 3). The patient was transferred to the general surgery ward on day 44 and discharged home on day 75 in stable condition. At 3 month follow-up, he remained asymptomatic and reported sustained abstinence from alcohol.

**Figure 3:**
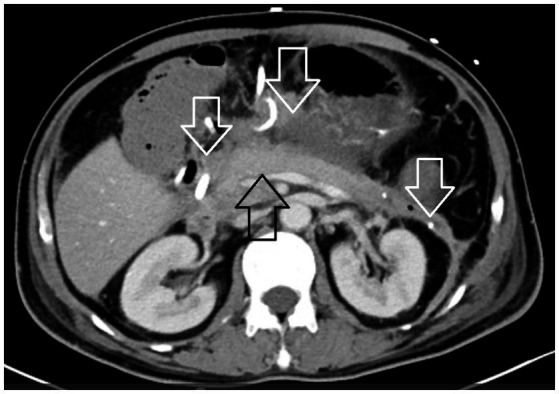
CECT day 47. Axial CECT image demonstrating near-complete resolution of peri-pancreatic fluid collections following laparoscopic necrosectomy and continuous postoperative lavage. Black arrow indicates minimal residual pancreatic necrosis. White arrow indicates substantial reduction in the previously noted walled-off necrosis.

## Discussion

This case highlights three interrelated challenges in SAP management: (1) confirmation of culture-proven IPN within the first week; (2) successful management of polymicrobial infection with CRAB and intrinsically carbapenem-resistant *E. faecium*; and (3) pragmatic use of a novel SUL-DUR-based regimen during CRRT.

### Early onset of IPN: challenging the conventional timeline

The first positive culture from peri-pancreatic fluid was obtained on day 7, which is earlier than the typical second-week onset described in classical literature (1–3). However, whether the infection had already been established before day 7 remains unknown. A literature search identified no prior case of IPN confirmed within the first 7 days with CRAB and *E. faecium* co-infection. Several factors may explain this accelerated timeline: invasive mechanical ventilation, persistent multi-organ failure, delayed enteral nutrition, and elevated admission pro-calcitonin (12, 13). Moreover, sustained intra-abdominal hypertension (IAP > 20 mmHg for over 10 days) creates a state of splanchnic hypoperfusion and is recognized as detrimental to gastrointestinal integrity (6), which may accelerate bacterial translocation from the gut lumen to the pancreatic bed.

Three CRAB isolates from sputum, peritoneal fluid, and blood had nearly identical anti-biograms (Table 2), raising the possibility of clonal dissemination. Although a pulmonary source was less likely given imaging and oxygenation, hematogenous seeding from respiratory colonization cannot be fully excluded. Molecular typing was not performed to confirm clonality. Regardless of the route, the first positive culture was obtained within the first week, showing that IPN can be documented earlier than traditionally thought. Clinicians should maintain a high index of suspicion for early IPN in SAP patients with MDRO colonization, persistent fever, and refractory ACS. We acknowledge that the exact onset of IPN may have been earlier than day 7, as no peri-pancreatic fluid was sampled before that time. Therefore, while this case demonstrates that culture-proven IPN can be documented as early as the first week, the precise timing of infection initiation remains uncertain.

**Table 2:** Antibiotic susceptibility of three carbapenem-resistant *Acinetobacter baumannii* isolates.

Antibiotic	Testing method	Sputum (d5)	Body fluid (d7)	Blood (d9)
Tigecycline	Disk diffusion	S (16 mm)	S (16 mm)	S (16 mm)
Amikacin	MIC	S (8 μg/mL)	S (16 μg/mL)	S (8 μg/mL)
Ampicillin/sulbactam	MIC	R (≥32 μg/mL)	R (≥32 μg/mL)	R (≥32 μg/mL)
Cefazolin	MIC	R (≥64 μg/mL)	R (≥64 μg/mL)	R (≥64 μg/mL)
Cefepime	MIC	R (≥64 μg/mL)	R (≥64 μg/mL)	R (≥64 μg/mL)
Ceftazidime	MIC	R (≥64 μg/mL)	R (≥64 μg/mL)	R (≥64 μg/mL)
Ceftriaxone	MIC	R (≥64 μg/mL)	R (≥64 μg/mL)	R (≥64 μg/mL)
Ciprofloxacin	MIC	R (≥4 μg/mL)	R (≥4 μg/mL)	R (≥4 μg/mL)
Gentamicin	MIC	R (≥16 μg/mL)	R (≥16 μg/mL)	R (≥16 μg/mL)
Imipenem	MIC	R (≥16 μg/mL)	R (≥16 μg/mL)	R (≥16 μg/mL)
Levofloxacin	MIC	R (≥8 μg/mL)	R (≥8 μg/mL)	R (≥8 μg/mL)
Tobramycin	MIC	R (≥16 μg/mL)	R (≥16 μg/mL)	R (≥16 μg/mL)
Trimethoprim-sulfamethoxazole	MIC	R (≥320 μg/mL)	R (≥320 μg/mL)	R (≥320 μg/mL)
Piperacillin	Disk diffusion	R (6 mm)	R (6 mm)	R (6 mm)
Cefoperazone/sulbactam	Disk diffusion	I (16 mm)	I (16 mm)	I (18 mm)
Meropenem	Disk diffusion	R (6 mm)	R (6 mm)	R (6 mm)
Minocycline	Disk diffusion	R (12 mm)	R (13 mm)	R (12 mm)
Eravacycline	Disk diffusion	S (15 mm)	S (15 mm)	Not tested
Sulbactam/durlobactam	Disk diffusion	S (24 mm)	S (23 mm)	Not tested

MIC, minimum inhibitory concentration (μg/mL); S, susceptible; I, intermediate; R, resistant. Criteria based on CLSI M100.

### Rationale for SUL-DUR plus tigecycline

There is no known synergistic interaction between SUL-DUR and tigecycline; their combination was chosen solely to provide coverage against both CRAB and *E. faecium*, rather than to enhance anti-CRAB activity. To our knowledge, this is the first reported case of SUL-DUR combined with tigecycline for polymicrobial IPN involving CRAB and *E. faecium*. The 2024 IDSA guidance strongly recommends combining SUL-DUR with a carbapenem for CRAB infections (7), based on the ATTACK trial which enrolled patients with mono-microbial CRAB infections (8). The trial did not address polymicrobial infections with intrinsically carbapenem-resistant organisms.

In our patient, antecedent *E. faecium* bacteremia made addition of a carbapenem clinically untenable. The intrinsic resistance of *E. faecium* to carbapenems is non-negotiable, driven by low-affinity PBP5 (9), with meropenem MICs consistently >8 mg/L. Switching to a carbapenem would have withdrawn coverage for a pathogen already in the bloodstream. We selected tigecycline based on its activity against enterococci (14), high intra-abdominal tissue penetration, and compatibility with CRRT.

We acknowledge the pharmacological limitations of this approach. Tigecycline is not the preferred first-line agent for *E. faecium* bloodstream infections due to its bacteriostatic mechanism of action and relatively low serum area under the curve (AUC). While tigecycline monotherapy is generally discouraged for primary bacteremia due to low serum concentrations, the high intra-abdominal burden of disease and the use of CRRT (which may mitigate some under-dosing risks) provided a unique therapeutic context. In our patient, tigecycline served as a suppressive backbone against enterococci until definitive source control (laparoscopic necrosectomy) could be achieved, while SUL-DUR provided bactericidal activity against the CRAB component. It is crucial to emphasize that the success of this strategy was critically dependent on definitive source control achieved through percutaneous drainage and subsequent laparoscopic necrosectomy. The rapid clearance of *E. faecium* and CRAB from repeat blood cultures provides anecdotal support for this strategy in the specific context of high-volume abdominal distribution.

### Management of refractory abdominal compartment syndrome

This case also illustrates the utility of neuromuscular blockade as rescue therapy for refractory ACS. Continuous rocuronium infusion (1.4 μg/kg/min) reduced IAP from 33 to 21 mmHg within 24 h, likely by increasing abdominal wall compliance (5, 15). This allowed safe initiation of enteral nutrition and bridged the patient to definitive surgical management.

### Limitations

Beyond those already discussed, this case has additional limitations. First, while IPN was microbiologically confirmed on day 7, the exact onset may have been earlier because peri-pancreatic fluid was not sampled before that time. Second, the favorable outcome with SUL-DUR plus tigecycline cannot be generalized without larger cohorts or randomized trials. Third, no therapeutic drug monitoring was available for SUL-DUR during CRRT; the q8h interval was based on ex vivo modeling (11), and clinical pharmacokinetic studies are urgently needed.

## Conclusion

This case adds to the emerging evidence that IPN can be documented within the first week of SAP in patients with persistent organ failure and sustained intra-abdominal hypertension, though the exact onset remains uncertain. Furthermore, this report addresses a critical gap in current antimicrobial guidance: the management of polymicrobial infections involving CRAB and intrinsically carbapenem-resistant *E. faecium*. The combination of SUL-DUR with tigecycline represents a rational, mechanism-based salvage regimen that preserves activity against both pathogens. Although tigecycline is not preferred for enterococcal bacteremia, its use as a suppressive backbone—coupled with SUL-DUR for bactericidal anti-CRAB activity and definitive source control—may be a viable strategy in this specific, high-stakes scenario. Given the increasing prevalence of polymicrobial infections with MDROs, clinical studies are urgently needed to define the optimal partner drug for SUL-DUR when a carbapenem is contraindicated, as current guidance leaves a significant gap.

### Patient perspective

After discharge, I stopped drinking completely and followed a low-fat diet. I can now perform daily activities without abdominal pain. I am deeply grateful for the multidisciplinary care that helped me recover.

## Data Availability

The original contributions presented in the study are included in the article/supplementary material, further inquiries can be directed to the corresponding author.
